# 1,4-Bis(iodo­meth­yl)benzene

**DOI:** 10.1107/S1600536809021151

**Published:** 2009-06-13

**Authors:** C. John McAdam, Lyall R. Hanton, Stephen C. Moratti, Jim Simpson

**Affiliations:** a Department of Chemistry, University of Otago, PO Box 56, Dunedin, New Zealand

## Abstract

The centrosymmetric title compound, C_8_H_8_I_2_, was prepared by metathesis from the dibromo analogue. In the crystal structure, weak C—H⋯I inter­actions link the mol­ecules into stacks down the *b* axis. The structure is further stabilized by short I⋯I contacts [3.8433 (2) Å], forming undulating sheets in the (101) plane.

## Related literature

For the synthesis, see: Moore & Stupp (1986 ▶); Kida *et al.* (2005 ▶). For related structures, see: Basaran *et al.* (1992 ▶); Fun *et al.* (2009 ▶); Jones & Kus (2007 ▶); Zhang *et al.* (2007 ▶). For applications of dihalo-*p*-xylenes in living radical polymerization processes, see: Samakande *et al.*, (2007 ▶); Asandei *et al.* (2008 ▶). For other polymer applications, see: Leir & Stark (1989 ▶); Hochberg & Schulz (1993 ▶). For additional applications of dihalo-*p*-xylenes, see: Le Baccon *et al.* (2001 ▶); Sobransingh & Kaifer (2006 ▶); Song *et al.* (2008 ▶); Au *et al.* (2009 ▶). For details of halogen⋯halogen inter­actions, see: Pedireddi *et al.* (1994 ▶) and for reference structural data, see: Allen *et al.* (1987 ▶).
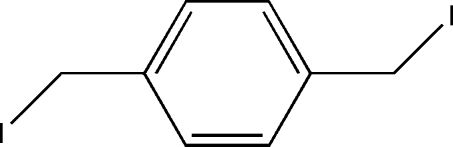


## Experimental

### 

#### Crystal data

C_8_H_8_I_2_
*M* *_r_* = 357.94
Monoclinic, 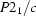

*a* = 9.0978 (3) Å
*b* = 4.5982 (2) Å
*c* = 11.2793 (3) Å
β = 99.808 (1)°
*V* = 464.96 (3) Å^3^
*Z* = 2
Mo *K*α radiation
μ = 6.69 mm^−1^
*T* = 89 K
0.21 × 0.15 × 0.03 mm

#### Data collection

Bruker APEXII CCD area-detector diffractometer
Absorption correction: multi-scan (*SADABS*; Bruker, 2006 ▶) *T*_min_ = 0.410, *T*_max_ = 0.818
8198 measured reflections
1674 independent reflections
1538 reflections with *I* > 2σ(*I*)
*R*_int_ = 0.026

#### Refinement

*R*[*F* ^2^ > 2σ(*F* ^2^)] = 0.013
*wR*(*F* ^2^) = 0.033
*S* = 1.06
1674 reflections
62 parameters
All H-atom parameters refined
Δρ_max_ = 0.51 e Å^−3^
Δρ_min_ = −0.51 e Å^−3^

### 

Data collection: *APEX2* (Bruker 2006 ▶); cell refinement: *APEX2* and *SAINT* (Bruker 2006 ▶); data reduction: *SAINT*; program(s) used to solve structure: *SIR92* (Altomare *et al.*, 1993 ▶); program(s) used to refine structure: *SHELXL97* (Sheldrick, 2008 ▶) and *TITAN* (Hunter & Simpson, 1999 ▶); molecular graphics: *ORTEP-3* (Farrugia, 1997 ▶) and *Mercury* (Macrae *et al.*, 2006 ▶); software used to prepare material for publication: *SHELXL97*, *enCIFer* (Allen *et al.*, 2004 ▶), *PLATON* (Spek, 2009 ▶) and *publCIF* (Westrip, 2009 ▶).

## Supplementary Material

Crystal structure: contains datablocks global, I. DOI: 10.1107/S1600536809021151/hb2998sup1.cif

Structure factors: contains datablocks I. DOI: 10.1107/S1600536809021151/hb2998Isup2.hkl

Additional supplementary materials:  crystallographic information; 3D view; checkCIF report

## Figures and Tables

**Table 1:** Hydrogen-bond geometry (Å, °)

*D*—H⋯*A*	*D*—H	H⋯*A*	*D*⋯*A*	*D*—H⋯*A*
C4—H42⋯I1^i^	1.02 (2)	3.12 (2)	3.9774 (16)	141.8 (16)

Symmetry code: (i) 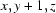.

**Fig. 1.**
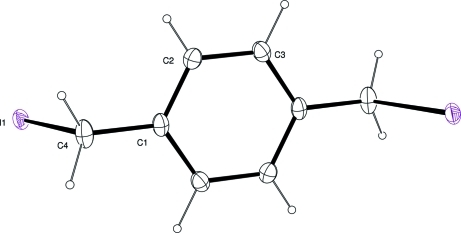
The structure of (I) with displacement ellipsoids for the non-hydrogen atoms drawn at the 50% probability level. Unlabelled atoms are generated by the symmetry operation (1–x, –y, 1–z).

**Fig. 2.**
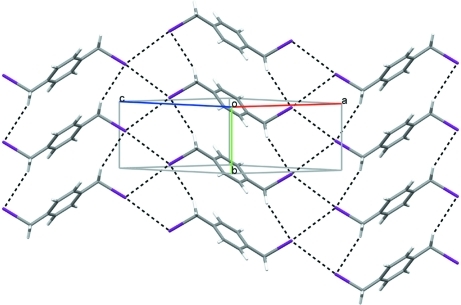
Crystal packing for (I) viewed down the b axis with hydrogen bonds and short I···I contacts drawn as dashed lines.

## supplementary crystallographic information

### Comment

Dihalo-*p*-xylenes are extensively used in polymer science (Leir & Stark, 1989; Hochberg & Schulz, 1993; Samakande *et al.*, 2007) and as a versatile synthon for –CH_2_—C_6_H_4_—CH_2_– connective units in other areas of chemistry (Le Baccon *et al.*, 2001; Song *et al.*, 2008; Au *et al.*, 2009). The bulk of this work utilizes the commercially available α,α'-dibromo-*p*-xylene, but the diiodo- derivative offers additional reactivity (Moore & Stupp, 1986; Sobransingh & Kaifer, 2006; Asandei *et al.*, 2008). Our interest in the title compound, (I), Fig. 1, is as one of the components in xylene bridged electroactive gels. In these, the chemical links are quaternary amines formed by reaction of the alkylhalogen termini with amine residues in the other gel component.

The molecule lies about an inversion centre located at the centroid of the benzene ring. The C1···C4 atoms lie in a plane (r.m.s. deviation 0.01 Å) and the C—C and C—I distances in the molecule are unremarkable (Allen *et al.*, 1987). This structure is the fourth in a series of XCH_2_C_6_H_4_CH_2_*X* molecules; *X* = F, II (Fun *et al.*, 2009); Cl, III (Basaran *et al.*, (1992); Br, IV (Jones & Kus, 2007; Zhang *et al.*, 2007). All four molecules are closely isostructural with only the C—halogen bond distance distinguishing them. Indeed the CH_2_C_6_H_4_CH_2_ fragment of the title compound overlays with corresponding portions of the related molecules with r.m.s. deviations, 0.032 Å for II, 0.013 Å for III and 0.007 Å for IV respectively (Macrae *et al.*, 2006).

In the crystal structure, weak C—H···I interactions and short I···I contacts, 3.8433 (2) Å, form undulating sheets in the 101 plane, Fig. 2. Each I atom interacts with two adjacent iodine atoms (symmetry operations 2 - *x*, -1/2 + *y*, 1/2 - *z* and 2 - *x*, 1/2 + *y*, 1/2 - *z*). The I···I contacts observed here fit with the type II designation of halogen···halogen interactions proposed previously (Pedireddi *et al.* 1994). The packing arrangement for I is closely similar to that observed for IV, (Jones & Kus, 2007; Zhang *et al.*, 2007). I, III and IV all crystallize in the space group P21/c with unit cells that differ only in a small but significant increase in volume as the size of the halogen increases.

### Experimental

The title compound was prepared by a combination of the methods of Moore & Stupp (1986) and Kida *et al.* (2005). Thus α,α'-dibromo-*p*-xylene (1.32 g, 5 mmol) was refluxed for 7 h with sodium iodide (2.25 g, 15 mmol) in acetone (25 ml). The solution was allowed to cool overnight, and the resulting yellow plates of (I) that developed were rinsed gently with water to remove sodium bromide and air dried. Confirmation of the metathesized (iodo) product was by microanalysis, mass spectroscopy and diagnostic tests. ^1^H and ^13^C NMR spectra are distinct from those of the dibromo precursor.

### Refinement

All H-atoms were located in a difference Fourier map and refined freely.

### Crystal data

**Table:**

C_8_H_8_I_2_	*F*(000) = 324
*M**_r_* = 357.94	*D*_x_ = 2.557 Mg m^−^^3^
Monoclinic, *P*2_1_/*c*	Mo *K*α radiation, λ = 0.71073 Å
Hall symbol: -P 2ybc	Cell parameters from 5590 reflections
*a* = 9.0978 (3) Å	θ = 2.3–33.0°
*b* = 4.5982 (2) Å	µ = 6.69 mm^−^^1^
*c* = 11.2793 (3) Å	*T* = 89 K
β = 99.808 (1)°	Rectangular plate, pale yellow
*V* = 464.96 (3) Å^3^	0.21 × 0.15 × 0.03 mm
*Z* = 2	

### Data collection

**Table:**

Bruker APEXII CCD area-detector diffractometer	1674 independent reflections
Radiation source: fine-focus sealed tube	1538 reflections with *I* > 2σ(*I*)
graphite	*R*_int_ = 0.026
ω scans	θ_max_ = 33.4°, θ_min_ = 3.7°
Absorption correction: multi-scan (*SADABS*; Bruker, 2006)	*h* = −13→13
*T*_min_ = 0.410, *T*_max_ = 0.818	*k* = −5→7
8198 measured reflections	*l* = −16→16

### Refinement

**Table:**

Refinement on *F*^2^	Primary atom site location: structure-invariant direct methods
Least-squares matrix: full	Secondary atom site location: difference Fourier map
*R*[*F*^2^ > 2σ(*F*^2^)] = 0.013	Hydrogen site location: inferred from neighbouring sites
*wR*(*F*^2^) = 0.033	All H-atom parameters refined
*S* = 1.06	*w* = 1/[σ^2^(*F*_o_^2^) + (0.0145*P*)^2^ + 0.1407*P*] where *P* = (*F*_o_^2^ + 2*F*_c_^2^)/3
1674 reflections	(Δ/σ)_max_ = 0.001
62 parameters	Δρ_max_ = 0.51 e Å^−^^3^
0 restraints	Δρ_min_ = −0.51 e Å^−^^3^

### Special details

**Table:**

Geometry. All e.s.d.'s (except the e.s.d. in the dihedral angle between two l.s. planes) are estimated using the full covariance matrix. The cell e.s.d.'s are taken into account individually in the estimation of e.s.d.'s in distances, angles and torsion angles; correlations between e.s.d.'s in cell parameters are only used when they are defined by crystal symmetry. An approximate (isotropic) treatment of cell e.s.d.'s is used for estimating e.s.d.'s involving l.s. planes.
Refinement. Refinement of *F*^2^ against ALL reflections. The weighted *R*-factor *wR* and goodness of fit *S* are based on *F*^2^, conventional *R*-factors *R* are based on *F*, with *F* set to zero for negative *F*^2^. The threshold expression of *F*^2^ > σ(*F*^2^) is used only for calculating *R*-factors(gt) *etc*. and is not relevant to the choice of reflections for refinement. *R*-factors based on *F*^2^ are statistically about twice as large as those based on *F*, and *R*- factors based on ALL data will be even larger.

### Fractional atomic coordinates and isotropic or equivalent isotropic displacement parameters (Å^2^)

**Table:**

	*x*	*y*	*z*	*U*_iso_*/*U*_eq_	
C1	0.62504 (16)	0.1521 (3)	0.47504 (13)	0.0138 (2)	
C2	0.63784 (17)	−0.0405 (3)	0.57245 (14)	0.0154 (3)	
C3	0.51429 (17)	−0.1903 (3)	0.59710 (14)	0.0154 (3)	
C4	0.75717 (18)	0.3189 (4)	0.45106 (15)	0.0190 (3)	
I1	0.880515 (9)	0.07951 (2)	0.331669 (8)	0.01438 (4)	
H2	0.729 (3)	−0.073 (4)	0.620 (2)	0.021 (6)*	
H41	0.841 (3)	0.354 (5)	0.524 (2)	0.031 (6)*	
H3	0.523 (2)	−0.330 (5)	0.6678 (19)	0.022 (5)*	
H42	0.733 (3)	0.509 (5)	0.405 (2)	0.021 (5)*	

### Atomic displacement parameters (Å^2^)

**Table:**

	*U*^11^	*U*^22^	*U*^33^	*U*^12^	*U*^13^	*U*^23^
C1	0.0142 (6)	0.0109 (6)	0.0180 (6)	−0.0017 (5)	0.0078 (5)	−0.0032 (5)
C2	0.0133 (6)	0.0159 (7)	0.0174 (7)	0.0007 (5)	0.0038 (5)	−0.0010 (5)
C3	0.0175 (6)	0.0126 (6)	0.0175 (6)	0.0006 (5)	0.0070 (5)	0.0012 (5)
C4	0.0191 (7)	0.0150 (7)	0.0258 (8)	−0.0039 (5)	0.0124 (6)	−0.0051 (6)
I1	0.01367 (5)	0.01628 (6)	0.01473 (5)	−0.00037 (3)	0.00680 (3)	0.00092 (3)

### Geometric parameters (Å, °)

**Table:**

C1—C3^i^	1.396 (2)	C3—C1^i^	1.396 (2)
C1—C2	1.401 (2)	C3—H3	1.01 (2)
C1—C4	1.489 (2)	C4—I1	2.1907 (15)
C2—C3	1.386 (2)	C4—H41	1.04 (2)
C2—H2	0.92 (2)	C4—H42	1.02 (2)
			
C3^i^—C1—C2	118.88 (13)	C1^i^—C3—H3	118.7 (12)
C3^i^—C1—C4	120.68 (14)	C1—C4—I1	111.52 (10)
C2—C1—C4	120.42 (14)	C1—C4—H41	116.4 (13)
C3—C2—C1	120.64 (14)	I1—C4—H41	100.5 (13)
C3—C2—H2	119.1 (14)	C1—C4—H42	114.9 (13)
C1—C2—H2	120.3 (14)	I1—C4—H42	101.8 (13)
C2—C3—C1^i^	120.48 (14)	H41—C4—H42	109.8 (19)
C2—C3—H3	120.9 (12)		
			
C3^i^—C1—C2—C3	−0.1 (2)	C3^i^—C1—C4—I1	−91.95 (15)
C4—C1—C2—C3	178.24 (14)	C2—C1—C4—I1	89.71 (15)
C1—C2—C3—C1^i^	0.1 (2)		

Symmetry codes: (i) −*x*+1, −*y*, −*z*+1.

### Hydrogen-bond geometry (Å, °)

**Table:**

*D*—H···*A*	*D*—H	H···*A*	*D*···*A*	*D*—H···*A*
C4—H42···I1^ii^	1.02 (2)	3.12 (2)	3.9774 (16)	141.8 (16)

Symmetry codes: (ii) *x*, *y*+1, *z*.
